# Thermomechanical Autovaporization (MFA) as a Deodorization Process of Palm Oil

**DOI:** 10.3390/foods11243952

**Published:** 2022-12-07

**Authors:** Bassem Jamoussi, Cherif Jablaoui, Amira K. Hajri, Radhouane Chakroun, Bandar Al-Mur, Karim Allaf

**Affiliations:** 1 Department of Environmental Sciences, Faculty of Meteorology, Environment and Arid Land Agriculture, King Abdulaziz University, Jeddah 21589, Saudi Arabia; 2 Valorgrain Company-Technology Department “MFA Technology”, 60220 Abancourt, France; 3 LaSIE (Laboratory of Engineering Sciences for Environment), La Rochelle University, 7356 UMR CNRS. Avenue Michel Crépeau, 17042 La Rochelle, France; 4 Department of Chemistry, Alwajh College, University of Tabuk, Tabuk 71421, Saudi Arabia

**Keywords:** palm oil, Multi-Flash Autovaporization (MFA), volatile compounds, deodorization, modeling, optimization, quality preservation

## Abstract

Throughout the vegetable oil industry, there is a focus on eradicating the volatile molecules affecting the aroma or taste of the crude oil, whether it is natural or derived from the extraction process. Refining aims to reduce these compounds to a level acceptable to the consumer. In addition, the famous conventional operation of deodorization calls for high levels of temperature depending on the boiling point used to remove the atmospheric pressure of each molecule. The process implies a vacuum level between 10 to 80 kPa absolute pressure, a temperature generally between 190 and 240 °C, and a duration of 2 to 3 h. These conditions necessarily (inevitably) lead to a decrease in the quality of refined oil. Recently, the application of the Multi-Flash Autovaporization “MFA” operation has shown the possibility of eradicating volatile molecules while adopting relatively low temperature and time levels. Despite the high boiling temperature of the volatile organic compounds (VOC), MFA leads to good efficiency in reducing VOCs and preserving oil quality. The main odorant compounds in the crude palm oil were E-2-Hexenal, heptanal, octanal, nonanal, and decanal. Specific literature can indicate precise boiling temperatures under atmospheric pressure. In addition, many experimental studies have explained the evolution of each molecule and shown how they depend on the operating parameters (inlet oil pressure from 200 to 450 kPa and from 5 and 30 s time of each cycle, and the number of cycles up to 7), and how the empirical mathematical models describe the MFA deodorization, estimate the efficiency of the whole process, and optimize the operating parameters. In this research, the thermodynamic data of absolute pressure volatility versus temperature was used to better identify the removal rate (up to around 87%) implied by an abrupt pressure drop to a vacuum of 5 kPa for *p* = 450 kPa, t = 25 s/cycle, and the number of cycles (C = 6). The safeguarding of the fatty acid profile illustrated the maintenance of the oil quality.

## 1. Introduction

The refining of vegetable oils generally involves successive operations of degumming, neutralization, bleaching, and deodorization [1]. Of these operations, deodorization plays an essential role in controlling evaporation and ensuring the elimination of a significant part of the volatile molecules responsible for odor and taste. The boiling level of these molecules under atmospheric pressure (thermodynamic aspect), and the duration of sweeping of the vapors generated in the surrounding environment (kinetic dimension), can be enormously detrimental to the oil quality and the nutritive content (fatty acids, antioxidant molecules, etc.).

There is still a growing need for vegetable oils, especially those containing bioactive components. The example of palm oil is the best from the point of view of richness in bioactive molecules with 500–700 ppm of carotenes, and 600–1000 ppm of tocopherols and tocotrienols [2]. It is practically the only vegetable oil that simultaneously contains the two fat-soluble antioxidants carotene and tocotrienols. In terms of quality and stability, palm oil is considered among the most stable oils, with good quality due to its richness in minor components such as polyphenols, phospholipids, and sterols [3]. In addition to its richness in phytonutrients such as 362–627 ppm of phytosterols, palm oil is distinguished by strong biological and antioxidant activity [4].

This aspect is critical as the conventional deodorization applied in the production chain of vegetable oils since the end of the 19th century involves a high-temperature level that can exceed 200 °C and an operation duration of a few hours (3–4 h). Vegetable oil deodorization requires a substantial specific intensification that simultaneously deepens the evaporation, reinforces the expulsion of the generated vapors far from the exchange surface, and thus reduces the reaction duration. The practical recognition of these elements can considerably reduce the degradation of the various vegetable oil molecules in question. However, such an extraction process requires removing the molecules responsible for the natural product odor and taste and residual extraction solvent traces. The efficiency of the deodorization operation must factor in the reduction rate of the molecules to promote their removal, the duration necessary for the process, and the energy consumed. It should also include good oil quality, preserve free fatty acids, and maintain the level and degree of preservation of antioxidant molecules, which are critical to vegetable oil’s sensory content and storage stability [5].

In the specific case of palm oil, conventional deodorization requires an extended period of high temperature. It entails several chemical reactions of oxidation, cis-trans isomerization, cyclization, and polymerization. Refs. [6,7] determined that some destruction of unsaturated fatty acids leads to the formation of the hazardous pollutants of glycidyl esters (GE) and 3-monochloropropane-1,2-diol. Refs. [8,9] showed that heating above 250 °C destructs the unsaturated fats in the oil and formation of new trans-fat compounds. These thermal conditions were classed as severe as they caused many chronic diseases, such as high blood pressure, cardiovascular disease, liver dysfunction, diabetes, etc. [10]. In the case of conventional deodorization operations, it is difficult to properly control the refining operational parameters at various ranks of processing oil pressure, temperature, time, and vacuum pressure. Thus, it would be challenging to manage the nutritional and sensory quality of refined oil [11]. Indeed, in the case of palm oil, [12] highlighted the tremendous effects of high temperature, which forms a considerable number of volatile molecules of aldehydes, alcohols, ketones, furans, aromatic compounds, acids, and esters. The critical point of oil heating operations was a temperature of 120 °C for creating volatile aldehydes and alcohols and 150 to 180 °C for developing ketones, furans, and acids [12]. None of these studies noted the evident implications of the time required for generating these molecules.

However, it is interesting to define an operation with high efficiency for removing or reducing volatile contaminants while reducing energy consumption and the environmental impact without a detrimental effect on the oil quality. The definition and design of a new deodorization operation must respect the composition of the oil considered, the type and model of the process as an evaporation pathway, and the operating parameters, including the treatment time [13]. Multi-Flash Autovaporization (MFA) is one of the most promising of the many new or emerging technologies. It comprises cycles with two successive stages: an active stage using pressure and temperature treatment, and a passive or tempering step occurring under a vacuum. The cycle duration remains low, leading to the easy execution of several repetitive cycles, each coupling the two phases of the active stage of high pressure/high temperature and the passive stage of reduced pressure. A study at the research scale will be able to analyze the impact of the various operating parameters to ensure a development to master, at the industrial scale, the MFA operation as an essential process in refining vegetable oil. This MFA deodorization operation is likely to become essential in the refining industry to improve the nutritional quality and taste/flavor of vegetable oil.

Deodorization is one of the most critical operations in the refining of vegetable oils. Due to the specific high-temperature and long-time operating conditions, as well as the low level of control, conventional methods cause the generation of trans fatty acids, glycerol polymers, and other undesirable by-products. The intensified deodorization operation of MFA requires an excellent thermodynamic equilibrium study to calculate the volatility of the various molecules concerned at each temperature [14]. At each temperature and for the concerned molecules, suitable software can define volatility as the absolute vapor pressure expressed in Pa, or relative volatility on a reference compound, such as water. The operating parameters of MFA are:

Inlet oil pressure P between 200 and 450 kPa.

Temperature between 120 and 148 °C.

Expansion rate (the pressure drop towards the vacuum and the releasing time).

Vacuum level (absolute pressure of 4 kPa).

Number of cycles.

The temperature level and duration of the alternative method of deodorization by MFA calls for more adequate conditions to eliminate odorous molecules while preserving the quality of the vegetable oil. Any experimental study must identify the effects of the operating parameters on the evolution of the various molecules concerned. A design of experiments (DoE) set can define, model, optimize, and control the growth of the molecules responsible for palm oil’s bad odor and taste, preserving the nutritional compounds and labeling the quality [15]. In terms of the physical processes, the pressure drop from the high-pressure/high-temperature treatment towards the vacuum directly influences the autovaporization of each of the volatile compounds. The short duration of heat treatment should positively impact the preservation of fatty acids and tocopherol antioxidant fractions present in palm oil.

## 2. Materials and Methods

### 2.1. Resources

The raw palm seeds were provided by the Carthage Grains Company. They were at 12.4% moisture content, and 11.78 g H_2_O/g db. The crude palm oil contained 200.08 µg of total tocopherol/g db (dry basis) and 582.1 µg/g of carotenoids fraction. Several pretreatment steps were undertaken; they involved crushing/grinding, solvent, and cold press extraction.

### 2.2. Chemical Products

The quality analysis of the crude palm oil referred to high-performance liquid chromatography (HPLC) and gas chromatography/mass spectrometry (GC-MS) analyses that establish the main composition in fatty acid profiles, tocopherol fractions, and volatile molecules and aromas. Polytechnic Institute UniLasalle (Beauvais-France) supplied the primary solvents, such as standard tocopherols and tocotrienols, toluene, acetonitrile, and chloroform, while Sigma-Aldrich provided the volatile standards, such as E-2-hexenal, heptanal, octanal, nonanal and decanal.

### 2.3. Experimental Methodology

**Figure 1:**
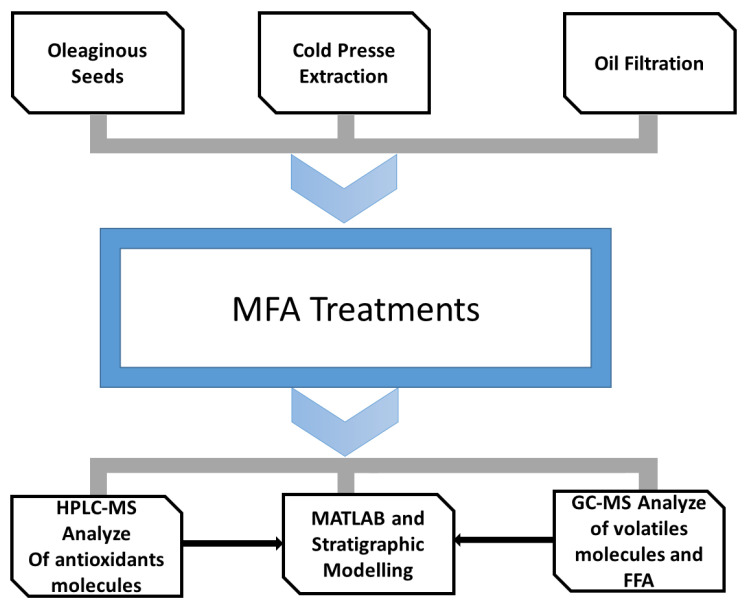
Schematic diagram of experimental study of palm oil deodorization by MFA (Multi-Flash Autovaporization).

The MFA process in the present research work was performed on oil obtained from pressing or solvent extraction. The gas chromatography (GC) and HPLC chromatographic analyses determined the quality of the oil in terms of the presence of volatile and non-volatile molecules, respectively (Figure 1).

The main objective of this study was to assess the impact of MFA operating parameters (hydraulic pressure and temperature of oil P+ and T+; cycle time t+, and the number of cycles N) on the operation deodorization performance and final oil quality. The short-duration/high-pressure treatment coupled with rapid and cyclic vacuuming is equivalent to the Instant Controlled Pressure Drop (DIC) treatment. Although the type of product treated (liquid for MFA and solid product for DIC) is different, the two processes are remarkably similar in autovaporization.

**Figure 2:**
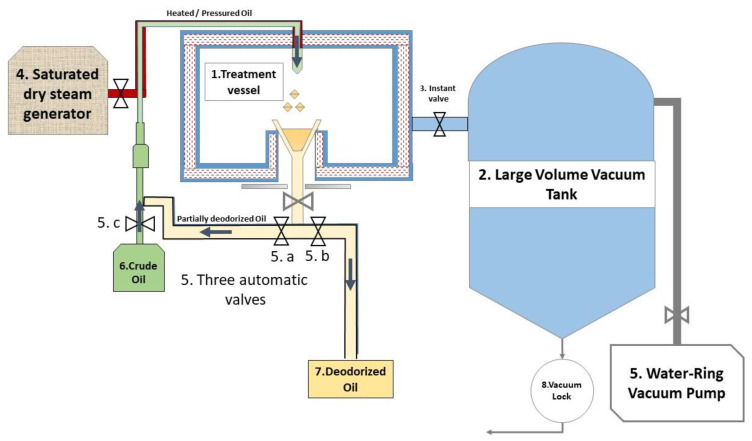
Schematic diagram of MFA industrial unit: 1/Vacuum vessel; 2/Large Volume Vacuum Reservoir; 3/Valve; 4/Saturated Steam Generator; 5/Automatic valves; 6/Crude oil tank; 7/Deodorized oil tank; 8/Vacuum lock; 9/Water-Ring Vacuum Pump.

**Figure 3:**
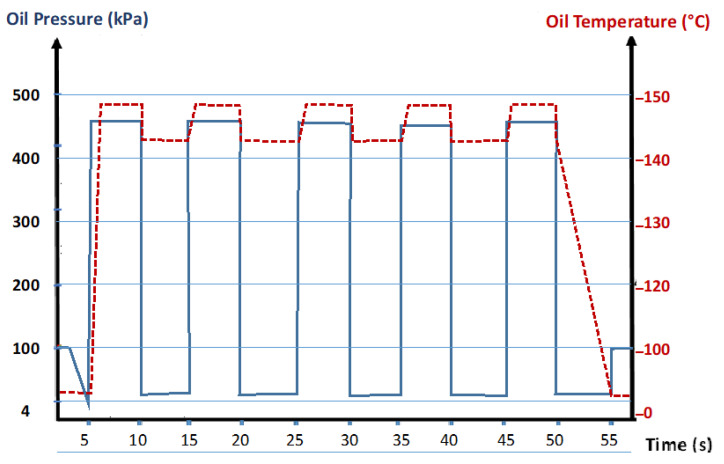
Pressure and Temperature Evolutions during MFA treatment (cycle time of 10 s; oil pressure of 450 kPa; oil temperature of 148 °C).

The MFA technology shown in Figure 2 involves spraying the oil in an airtight vacuum vessel (1). The pressure profile in Figure 3 shows that the oil pressure fluctuates between the high-oil pressure rated P^+^ and the low-pressure rated P^−^. In the first cycle, and to establish an initial high temperature/high pressure of oil, the spraying into the vacuum vessel was associated with the partial autovaporization of volatile molecules. The oil undergoes a cyclic pressure variation from the high temperature (up to 148 °C)/high-pressure stage (up to 450 kPa), assuring compression towards the vacuum stage (4 kPa). This abrupt spraying provides an instantaneous decompression phase (dP/dt > 5 MPa s^−1^). Three automatic valves (5) connect the crude or partially deodorized oil to the treatment vacuum vessel (1). The oil undergoes successive MFA treatment cycles by closing valves (5.b) and (5.c). The closing of valves (5.a) and (5.c) marks the end of the treatment; oil reaches the deodorized tank (7), and the crude oil begins a new MFA treatment.

Before reaching the nozzle, the oil pressure was between 200 and 450 kPa. Independently, various ways of heating hot air, electrical resistance, or high saturated steam pressure may heat the product, usually up to 148 °C, for a time noted t+ of about 10 s. The surface temperature should demarcate the value $P_{i}^{+}$ of the partial vapor pressure of each volatile compound. The spraying towards the vacuum assures an abrupt decompression toward a vacuum (pressure of 5 kPa). This path reduces by α the ratio of molecules removed from each vapor compound (Equation (1)):

(1) $$\alpha =\frac{P_{i}^{+}-P_{i}^{-}}{P_{i}^{+}}=\frac{P^{+}-P^{-}}{P^{+}}\approx \frac{325\;\mathrm{kPa}-4\;\mathrm{kPa}}{325\;\mathrm{kPa}}\approx 98.8\%$$

Thus, the principal response was the concentrations of volatile molecules, fatty acids, and antioxidants in the oil. The calibration curves of the standard concentrations relate to the gas chromatographic GC and liquid HPLC results for each volatile and non-volatile molecule (primary fatty acids and tocopherols), respectively.

Various MFA treatments according to a well-defined experimental plan were carried out after the cold pressing and solvent extractions. The results compared the concentrations of different volatile and non-volatile molecules.

### 2.4. Deodorization by Multi-Flash Autovaporization MFA Process

The scientific basis for and previous applications of the MFA approach guarantee high process performance and liquid quality maintenance. The hydro-thermomechanical effects of high-pressure/high-temperature spraying till 5 kPa of absolute pressure approximately following rapid heating allows for a repeatable MFA processing cycle [15].

Figure 3 shows the MFA processing cycles. The oil passing in the heating/compressing zone achieved a high temperature and high pressure up to 148 °C and 450 kPa, respectively; they remained constant for a few seconds before causing, by spraying, a sudden decompression toward the vacuum of circa 4 kPa. The drop in total pressure induced an autovaporization of the volatile molecules.

As described in Figure 2, the impact of the MFA treatments depends on the processing conditions, with a temperature ranging from 120 to 148 °C for a pressure ranging from 200 to 450 kPa, and a total time not exceeding 2 min [16]. The intrinsic properties of the various product compounds should define the operating parameters. The spraying in the vacuum vessel of around 4 kPa results in a pressure-drop rate DP/Dt of 5 MPa s^−1^.

It is worth noting the impact of successive instantaneous relaxations on the vacuum, which is at the origin of the autovaporization phenomenon. They normally correspond to partially removing the gaseous molecules in the same total pressure ratio.

It is essential to optimize the amount of vapor sweeping out P_i_ for each volatile compound during an MFA cycle to better control the phenomenon of deodorization and modification of oil properties.

### 2.5. Assessments and Characteristics

#### 2.5.1. Identification of Volatile Compounds of the Palm Oil

Headspace-solid phase microextraction (HS-SPME) is one of the main techniques for identifying and assessing the volatile compounds of oil. Refs. [17,18] used it to analyze the aromas of various natural products. HS-SPME involves a fiber coated by polydimethylsiloxane (PDMS, 100 μm) for enthralling the volatile molecules. The measurements of oil aromas consisted of manually placing a 5-mL sample of oil in the 10-mL headspace, holding it at 40 °C for 10 min to reach equilibrium, and then stopping this step 30 min later. Thermal desorption of this fiber lasts 5 min in a GC (gas chromatograph) without fractionation. Each SPME injection required 20 min for neutralization or reconditioning. It can then receive a new sample in the headspace and resume the GC-MS measurement [19,20].

#### 2.5.2. Chromatographic Measurements

Agile 19091S-433 gas chromatography (Kyoto, Japan) set with an HP-5MS column (30 m × 350 µm × 0.25 µm) characterizes the qualitative aspect of palm oil. The aim was to determine the profile of the prominent fatty acids defined in the percentage of FFA (free fatty acids). The method adopted consisted of increasing the oven temperature from 155 to 230 °C, and then stabilizing it at 240 °C for an analysis time of 50 min and 0.37 m/s as carrier gas flow. Mixing 15 µL of crude palm oil with 20 µL of TMH (Tolbutamide Methyl-Hydroxylase) and 1 mL of a solution (50/50) of chloroform/methanol took place. The system automatically inoculated 1 µL of each sample in a 1/200 fragmented manner.

The identification and quantification of the free fatty acids involved a combination of the computer software Gaz Chromatography Library, retention times, and concentration of standard compounds.

The determination of the α, β, and γ tocopherol fractions took place using Shimadzu system liquid chromatography (HPLC) (Kyoto, Japan) with an FL/FR-10AXL sensor with a 250 × 4.6 mm × 5 µm Altimma RPC-18 separation column (Associates Inc., Roseville, CA, USA).

The temperature had to alleviate at 25 °C and the fluorescence device set at 298 and 344 nm, before performing an automatic injection of 25 µL of the sample with an acetonitrile/methanol (75/25) mobile phase at 1 mL/mm flow rate. Retention times compared to injected standards of known concentrations allow identification of the tocopherol α, β, and γ fractions.

#### 2.5.3. Using Conditions of the GC-MS

The GC-MS chromatography system (Agilent 7890A. Santa Clara, CA 95051; USA) with an HP-5MS (5% Phenyl Methyl Siloxane) column (30 m, a diameter of 0.25 mm, and a thickness of 0.25 µm) identified and quantified the main volatile molecules of crude palm oil. Helium was used as a carrier gas conveyed at 1 mL/min as a flow rate with 5 min as a desorption time. The oven temperature increased from 40 to 250 °C in 42 min and the analysis time was 45 min. The identification of the volatile compounds involved matching the mass spectra with those of each reference compound in the mass spectra (M.S.) library [21].

#### 2.5.4. Design of Experiments (DoE)

The study and statistical optimization of the impact of the MFA operating parameters to deodorize crude palm oils led to the use of a 2-parameter, 5-level central composite CCD-DoE design. Refs. [22,23] identified the ranges and optimized processing parameters for autovaporization of odor and taste. Thus, the inlet oil pressure P from 200 to 450 kPa, the time t from 10 to 30 s/cycle, and the number of cycles C from 1 to 7 cycles were the independent variables and ranges retained of DoE of MFA deodorization after preliminary tests to keep constant the vacuum pressure at 4 kPa and the oil inlet temperature between 120 and 148 °C (Table 1).

This experimental study must identify the effects of the operating parameters on the evolution of the various molecules concerned. The CCD-DOE model was an integral part of the surface response mythology. One of the greatest advantages of this type of optimization model is that it is accurate and does not require a three-level factorial experiment. In this design, the curvature estimation can be improved by adding a group of additional “star points”.

The GC-MS chromatographic measurements of the different crude and differently treated MFA oils led to the identification of the concentration of each volatile molecule in terms of the reduction percentage as the crucial response (dependent variable). Statistical analysis of the experimental results through the response surface methodology (RSM) performed by STATGRAPHICS^®^ plus for Windows^®^ software (Statgraphics centurion XV, StatPoint Technologies, Inc., Rockville, MD, USA) considered each response scored (Y) versus the independent variables X_i_. ANOVA (Analysis of variance) based on a 5% probability *p*-value (*p* < 0.05) was used to determine the best differences between the parameters. The Pareto chart using horizontal bars was used to indicate the significance level of the parameter’s impact.

A second-order empirical equation was used to express the variation of the response (Y) versus the independent variables and define a multi-criteria optimization of the treatment. Its regression coefficient R was used to verify the model and quantify the fitting level.

## 3. Results and Discussion

### 3.1. Profile of Volatile Molecules as a Function of MFA Processing Parameters

GC-MS analyses were used to define the performance of MFA technology as a deodorization process for various palm oil samples. The reference was palm oil extracted by organic solvent using the Randall Velp-148 technique (6 h at 55 °C). The chromatographic analysis of the extracted oil showed main peaks for five volatile molecules. These results were consistent with those found by [24].

Table 2 shows the presence of five major molecules in the crude palm oil: E−2-hexenal, heptanal, octanal, nonanal, and decanal. The percentages from the integration of the identified significant peaks were 13.27, 15.22, 12.05, 35.61, and 11.45%, respectively.

### 3.2. Multi-Flash MFA Autovaporization as a Deodorization Process

Equation (2) determines each molecule’s reduction rate (RR) depending on the concentrations calculated from the peak areas and presents it as a function of the MFA variation of processing parameters of pressure (P), processing time per cycle (t), and cycles C (Table 3).

(2) $$RR\left(\%\right)=\frac{\left(area\;control\;sample-area\;treated\;sample\right)}{area\;control\;sample}\%$$

Table 3 shows that the percentage reduction of the five main volatile molecules in the different MFA treatments compared to the crude palm oil depended significantly on the variation of the MFA operating parameters. The central interpretation depended on the number of cycles. Thus, at P = 325 kPa and t = 18 s per cycle, E-2-hexenal was reduced by 12.0 and 84.0%, for C = 1 and 7, respectively. For these same treatment levels, the impact of the number of cycles was critical (see MFA 21 and MFA 3). The effect of P and t also occurred but to a lesser degree.

The highest reduction percentages reached 87.3, 78.7, 75.1, 62.4, and 65.1% for E-2-hexenal, heptanal, octanal, nonanal, and decanal, respectively, for 6 cycles at 399.3 kPa and 25 s/cycle. However, the maximum reduction for nonanal was 72.5% for 6 cycles at 399.3 kPa at 10 s/cycle.

**Figure 4:**
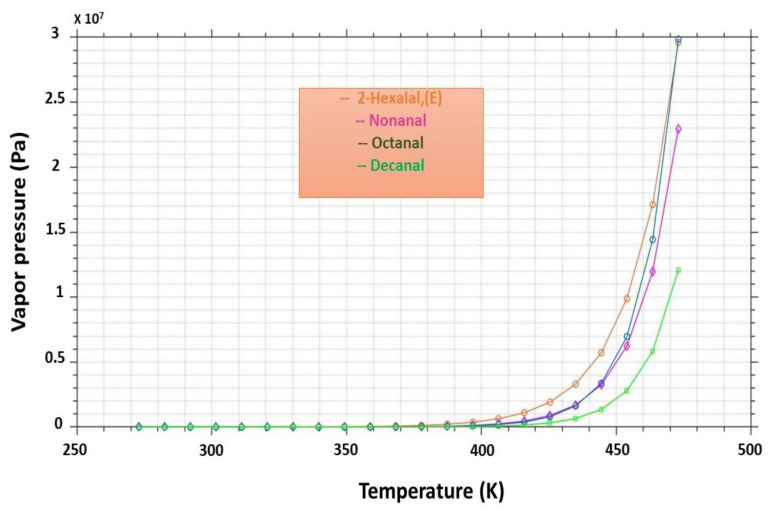
Variation of vapor pressure “volatility” for the major volatile’s molecules in the crude palm oil versus temperature “applied pressure” during MFA treatment.

The deodorization efficiency and the elimination of the major volatile molecules are a function of the operating conditions; however, both the chemical and thermodynamic properties of each molecule or aroma play an important role in the efficiency of this process. As described in Figure 4, and to better explain and discuss our experimental results, we studied the variation of the volatility “vapor pressure” of each molecule as a function of the temperature “applied pressure”. This predictive and detailed study of the variation of the volatility of the molecules was based on [25] modeling of mister x using MATLAB programming.

### 3.3. Statistical Analysis

Table 1 defines the impacts of the MFA operating parameters P, t, and C obtained from the 5-level composite-centered design CC-DoE (BBD). The statistical analysis concerning the deodorization efficiency depended on the statistical parameters (ANOVA, P-value) in Table 4 and allowed the optimization of the whole operation.

Table 4 shows the empirical models issued from the RSM of DoE, while Table 4 shows that the most significant probability corresponded to the number of cycles C. The fitted models validated the different significant impacts of the operating parameters (as the independent variables) on the dependent variables. The statistical analysis revealed a highly significant impact (*p* < 0.05) of the number of cycles (C), as well as the treatment time per cycle (t) and the input oil pressure (P) on the reduction rates (RR) of the volatile molecules considered.

**Figure 5:**
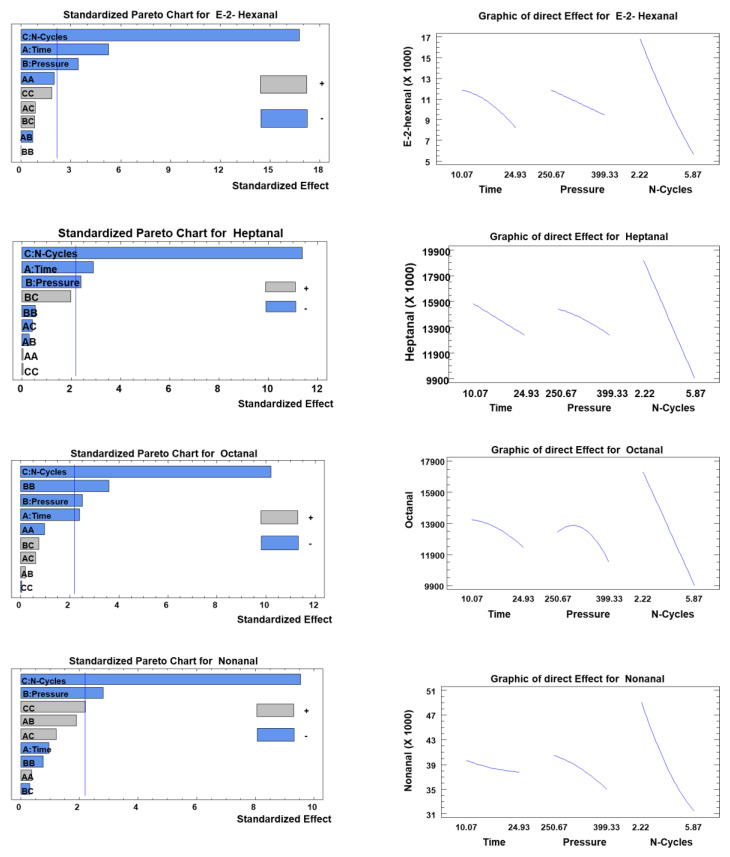

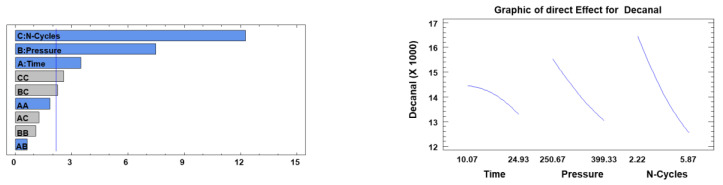
Standardized Pareto plots of MFA operating parameters t, P, and C on the deodorization of each volatile compound.

**Figure 6:**
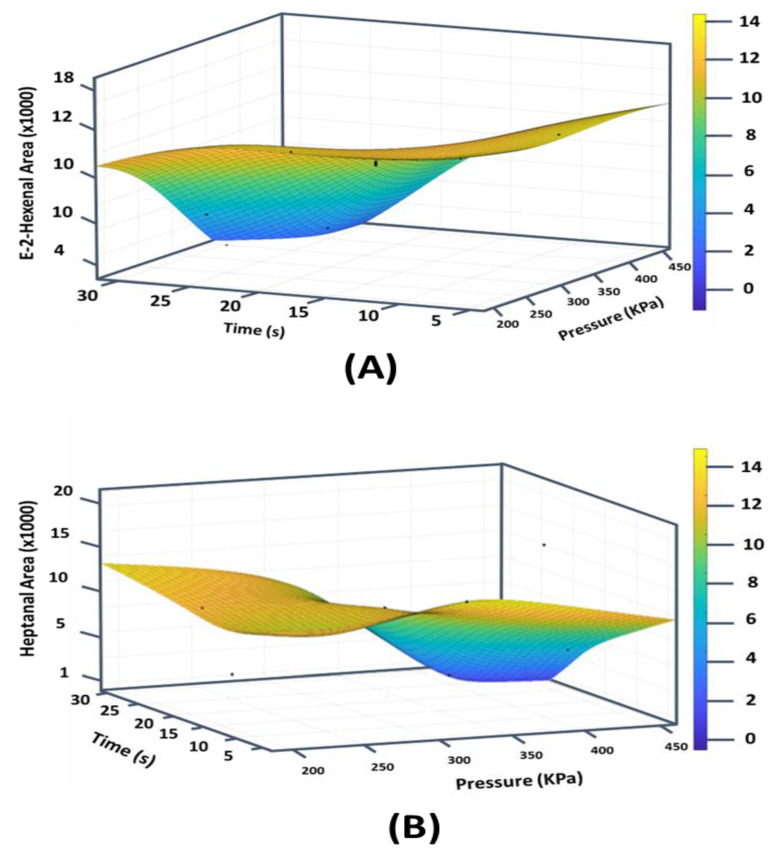

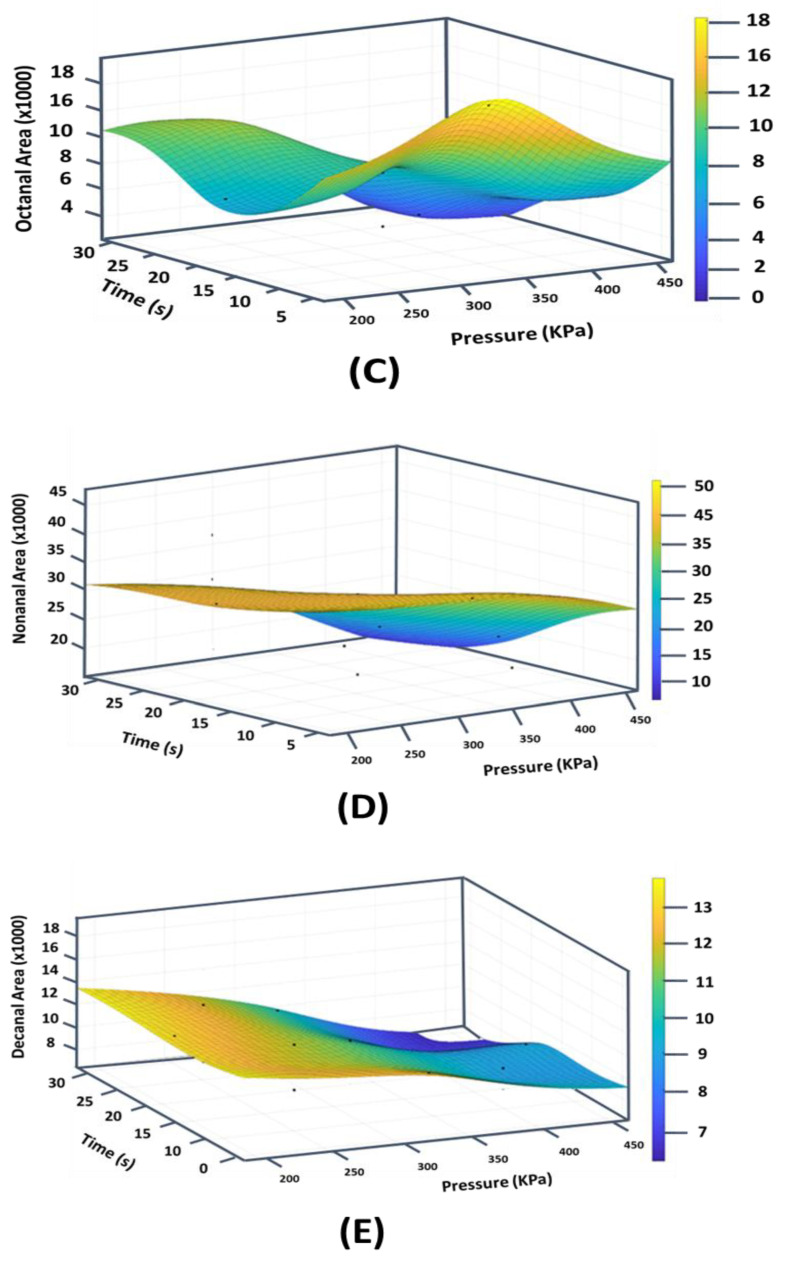
Response surface curves of residual aroma compounds versus treatment time and pressure for several cycles C = 6 of palm oil ((**A**–**E**) respectively for E-2-hexenal, heptanal, octanal, nonanal, and decanal).

The main trends (Figure 5) of the effects of the MFA P, t, and C on the aroma concentrations in MFA-treated palm oil confirmed the mainly linear effect of the operational parameters. The quadratic effects of C, P, and t allowed statistical optimization of the AMF operating parameters (Figure 6).

Statistical analysis showed that the most significant effect (*p* < 0.05) was for C and then, at a lower level, for P, and finally t, on the concentration of the five volatile molecules.

### 3.4. Optimization of AMF Pretreatment

The second-order mathematical equation (Table 5) reveals the statistical analyses of RR of the volatile molecules in the MFA-treated palm oil compared to the crude palm oil as a function of P, t, and C. This expression was the means of optimizing the MFA operating parameters.

Table 5 and Figure 5 represent the statistical optimization of the reduction ratio (RR) (%) and the response surface curves of the residual composition of molecules E−2-hexenal, heptanal, octanal, nonanal, and decanal, respectively.

The most remarkable results to emerge were that the different pressure gradients during the MFA technology allowed an acceleration of the evaporation of molecule flavors. As indicated by the work of [14], this was also supported by the chemical properties of the molecules and mainly by the thermodynamic properties, such as the vapor pressure or volatility of each molecule.

In the context of research work on the optimization of deodorization processes as a function of the variation of the operating conditions as described by the equation or law of “Dalton” and “Raoult” [26], Table 6 lists the level and MFA operating parameters of multi-criteria optimization of the reduction ratio (RR) (%). The optimums of the operating parameters pressure/temperature P/T = 450 kPa/148 °C, processing time t = 30.0 s/cycle, and number of MFA cycles C = 7, were for the optimum reduction ratios (RR) of 91.1, 98.5, 99.3, and 88.2% for E-2-hexenal, heptanal, octanal, and nonanal, respectively. As indicated in research works by [27] on soybean oil deodorization, MFA technology applied with optimized conditions shows a high efficiency also on crude palm oil as the optimum reduction ratio (RR) of decanal was 81.5% for the pressure/temperature, processing time, and number of cycles optima of 200 kPa/120 °C, 30.0, and 7, respectively. Based on the research work of Liu et al. (2021), the results of this study clearly indicate the disadvantage of the high-temperature (240–250 °C) conventional deodorization process during 2–3 h versus the advantage of MFA as an innovative process in the deodorization process without thermal deterioration of the product.

#### Impact of MFA Treatments on Palm Oil Quality

**Figure 7:**
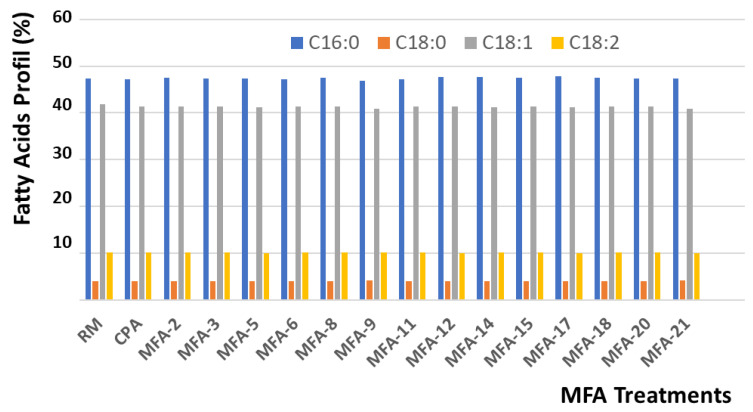
Variation Fatty Acids Profile versus MFA processing parameters.

Chromatographic measurements quantified and identified the saturated and unsaturated fatty acids in palm oil treated under various MFA operating parameters. Figure 7 illustrates the preservation of palm oil quality by keeping constant the concentration and was similar to the profile of principal fatty acids such as palmitic acid (C16:0), (C18:0), oleic acid (C18:1), and linoleic acid (C18:2).

**Figure 8:**
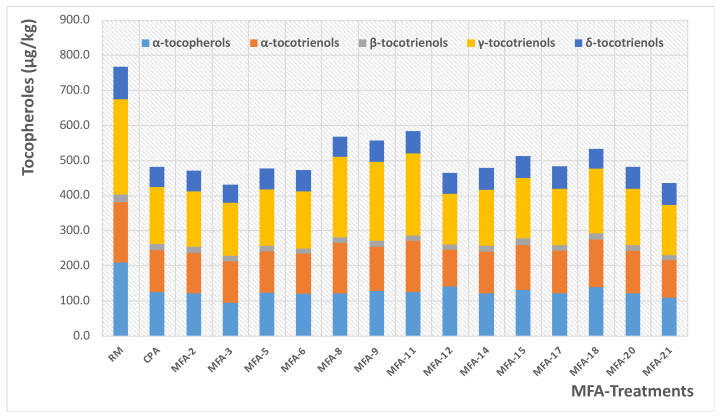
Variation of the antioxidant activity versus the MFA conditions.

Figure 8 shows the slight variation in the content of the different fractions of α tocopherols, α, β, γ, and δ tocotrienols, according to the various MFA operating conditions. A comparative study confirmed an overall decrease of close to 31% of the indicated molecules. An analysis of Figure 6 and Figure 7 shows highly satisfying preservation of the oil quality, whatever MFA deodorization parameters were applied.

Optimized MFA treatment preserved the quality of the palm oil and did not affect its biochemical composition. As long as the heating required for autovaporization was short, it was possible to preserve the fatty acids. The reduction in aroma correlated with the instant pressure drop toward a vacuum (5 kPa). Refs. [28,29] proved that the high-temperature/short treatment time of optimized MFA involved deodorization coupled with significant preservation of the main biochemical composition, even when applied on heat-sensitive molecules.

In comparison with the work of [30] and recently [31,32], this current research study shows that the application of MFA technology in the process of the deodorization of vegetable oil allows preservation of quality and conservation of the typical flavor that is an attractive quality.

## 4. Conclusions

As an alternative to the conventional deodorization technique, the innovative MFA deodorization method was applied to palm oil. The combination of an abrupt pressure drop to the vacuum from an initial hydraulic pressure below 450 kPa, a temperature between 120 and 148 °C allowed the removal or reduction of undesirable volatile molecules. The multi-flash aspect of the successive MFA cycles acts according to conditions that are dependent on the volatility of these molecules without requiring a high-temperature level with a relatively short total duration. The DoE achieved a multi-criteria optimization of the AMF process at 450 kPa, 30 s/cycle with 7 cycles, leading to a reduction of the volatile molecules concerned from more than 82 up to 99%, with preservation of the fatty acids and a low decrease of the antioxidants.

MFA deodorization at P = 450 kPa for C = 7 cycles of 30 s/cycle led to a reduction of octanal by 99%. GC/MS chromatographic analyses indicated that palmitic acid (C16:0), oleic acid (C18:1), linoleic acid (C18:2), and linoleic acid (C18:3) remain unchanged. In addition, α tocopherols, and α, β, ϒ, and δ tocotrienols showed slight changes in composition.

Savings in terms of the refining process, time, and energy imply the sustainability of the developed treatment process. The finding of this work on the MFA concept demonstrates the significant potential of combining low temperature, short cycle times, and a large pressure gradient generated by the instant pressure drop within the new deodorization approach.

Thermomechanical multi-flash autovaporization (MFA) is a highly performing deodorization method for palm oil. Compared to conventional methods, the adequately optimized MFA is more effective, implies higher nutritional content, and has lower energy consumption. Further research is necessary to better optimize and industrialize the MFA as a new deodorization process for vegetable oil refining.

## Figures and Tables

**Table 1:** MFA treatment parameters for the deodorization of crude palm oil.

Coded Values	−α	−1	0	1	+α
**Treatment time t (s/cycle)**	5	10.07	17.50	24.93	7.43
**Inlet oil pressure P (kPa)**	200	250.67	325	399.33	450
**Number of cycles C**	1	2	4	6	7

**Table 2:** Profile of the main volatile organic compounds of fresh crude palm oil.

Molecules	Volatile Compounds in Crude Palm Oil				
	E−2-Hexenal	Heptanal	Octanal	Nonanal	Decanal
**Linear Retention Time (LRI)**	769	1104	1204	1350	1204
**Peak Area RM-1**	22,985	26,255	19,876	61,589	20,326
**Peak Area RM-2**	24,015	25,963	22,405	62,562	19,971
**Peak Area RM-3**	22,966	28,029	21,253	63,601	20,072
**Profile (%)**	13.27	15.22	12.05	35.61	11.45
**Standard deviation**	462.0	853.3	868.0	678.0	135.3
**Average**	23,322	26,749	21,178	62,584	20,123
**Coefficient of variance**	0.020	0.032	0.041	0.011	0.007

**Table 3:** DoE reduction rate (RR) of the main volatile molecules versus MFA treatment parameters.

DoE Trials	t Time (s/cycle)	P Pressure (kPa)	C Cycles	Reduction Ratio RR (%)				
				E−2-Hexenal	Heptanal	Octanal	Nonanal	Decanal
**RM**	0	0	0	1	1	1	1	1
**CPA**	18	325.0	4	56.7	51.3	48.7	57.6	55.1
**MFA-2**	30	325.0	4	77.8	55.6	56.9	57.2	58.0
**MFA-3**	18	325.0	7	84.0	77.8	70.7	67.7	61.0
**MFA-5**	18	450.0	4	67.1	64.4	65.4	69.0	61.6
**MFA-6**	18	200.0	4	42.4	48.0	56.0	48.7	43.6
**MFA-8**	25	250.7	6	86.6	74.2	68.5	69.0	60.7
**MFA-9**	10	250.7	2	22.8	34.3	27.9	40.7	39.6
**MFA-11**	25	250.7	2	34.1	35.8	50.6	51.3	45.2
**MFA-12**	10	399.3	6	81.4	75.3	69.4	72.5	60.2
**MFA-14**	10	399.3	2	25.0	32.7	41.9	44.1	49.1
**MFA-15**	10	250.7	6	75.4	69.9	65.4	61.2	59.2
**MFA-17**	5	325.0	4	44.7	39.6	45.5	54.2	54.7
**MFA-18**	25	399.3	6	87.3	78.7	75.1	62.4	65.1
**MFA-20**	25	250.7	2	28.1	34.0	28.5	46.0	44.9
**MFA-21**	18	325.0	1	12.0	23.1	25.5	34.4	40.1
**Standard deviation**				26.56	19.21	16.53	11.47	8.63
**Average**				51.65	49.73	49.80	52.30	49.95
**Coefficient of variance**				0.51	0.39	0.33	0.22	0.17

**Table 4:** Statistical data of DoE reduction rate (RR) versus MFA treatment parameters using RSM (response surface methodology).

MFA Treatments	E−2-Hexenal		Heptanal		Octanal		Nonanal		Decanal	
Model	F-Value	Proba	F-Value	Proba	F-Value	Proba	F-Value	Proba	F-Value	Proba
**T**	73.43	0.0001	11.62	0.0052	5.46	0.0375	7.59	0.0174	0.11	0.7444
**P**	16.82	0.0015	12.63	0.0041	8.59	0.0126	6.17	0.0287	5.38	0.0388
**C**	498.22	0.0002	368.5	0.0001	60.86	0.0002	124.32	0.0001	75.36	0.0003
**T^2^**	7.16	0.0202	0.76	0.4008	4.06	0.067	0.04	0.8545	0.1	0.762
**TP**	3.36	0.0918	1.78	0.2075	1.1	0.3156	0.12	0.7331	2.17	0.1666
**TC**	3.13	0.102	1.3	0.2766	0.12	0.7304	0.66	0.4315	3.76	0.0765
**P^2^**	0	0.9932	10.51	0.0071	7.83	0.0161	3.56	0.0834	0	0.9506
**PT**	1.21	0.2934	0.1	0.7519	1.75	0.2111	1.49	0.2457	0.28	0.6071
**C^2^**	11.33	0.0056	0.31	0.5868	0.13	0.7219	0.98	0.3411	5.54	0.0365
**R^2^**	98.42		97.65		90.69		93.38		90.45	
**R^2^_-Adj_**	97.24		95.88		83.71		88.41		83.29	

**Table 5:** RSM statistical analyses of MFA treatment data.

Model	Molecule Area				
	E−2-Hexenal	Heptanal	Octanal	Nonanal	Decanal
**Constant**	30,673	12,137	8578	37,563	28,559
**P**	−3.9	89.5	106.2	153.8	−41.4
**T**	282	−71	−201	−839	−120
**NC**	−5980	−2433	−2924	−4408	−2733
**P × T**	−1.09	−0.83	−1.12	0.69	1.24
**P × NC**	2.46	−0.76	5.33	−9.12	1.68
**T × NC**	38.6	26.0	−13.8	59.2	−60.1
**P^2^**	−0.0003	−0.1293	−0.1923	−0.2403	0.0034
**T^2^**	−10.23	3.48	13.85	2.39	−1.68
**NC^2^**	212	−37	−41	208	211
**R^2^**	98	98	91	93	90

**Table 6:** Optimization of MFA processing parameters.

Facteur Area	Control Value	Optimum Value	Optimum Reduction Ratio RR (%)	Pressure (kPa)/Temperature ( °C)	Time (s)	Numbers of Cycles
**E-2-Hexenal**	*23,322*	2083.38	91.1%	450 kPa/148 °C	30.0	6.9
**Heptanal**	*26,749*	408.953	98.5%	450 kPa/148 °C	29.0	7.0
**Octanal**	*21,178*	154.464	99.3%	450 kPa/148 °C	28.9	7.0
**Nonanal**	*62,584*	7374.9	88.2%	450 kPa/148 °C	22.7	7.0
**Decanal**	*20,123*	3717.75	81.5%	200 kPa/120 °C	30.0	7.0

## Data Availability

Data is contained within the article.
